# An Evaluation of Voluntary Varicella Vaccination Coverage in Zhejiang Province, East China

**DOI:** 10.3390/ijerph13060560

**Published:** 2016-06-03

**Authors:** Yu Hu, Yaping Chen, Bing Zhang, Qian Li

**Affiliations:** Institute of Immunization and Prevention, Zhejiang Provincial Center for Disease Control and Prevention, Hangzhou 310051, China; husix@163.com (Y.H.); ypchen@cdc.zj.cn (Y.C.); bzhang@cdc.zj.cn (B.Z.)

**Keywords:** varicella vaccine, immunization information system, coverage, birth cohort

## Abstract

*Background*: In 2014 a 2-doses varicella vaccine (VarV) schedule was recommended by the Zhejiang Provincial Center for Disease Control and Prevention. We aimed to assess the coverage of the 1st dose of VarV (VarV_1_) and the 2nd dose of VarV (VarV_2_) among children aged 2–6 years through the Zhejiang Provincial Immunization Information System (ZJIIS) and to explore the determinants associated with the VarV coverage. *Methods*: Children aged 2–6 years (born from 1 January 2009 to 31 December 2013) registered in ZJIIS were enrolled. Anonymized individual records of target children were extracted from the ZJIIS database on 1 January 2016, including their VarV and (measles-containing vaccine) MCV vaccination information. The VarV_1_ and VarV_2_ coverage rates were evaluated for each birth cohorts. The coverage of VarV also was estimated among strata defined by cities, gender and immigration status. We also evaluated the difference in coverage between VarV and MCV. *Results*: A total of 3,028,222 children aged 2–6 years were enrolled. The coverage of VarV_1_ ranged from 84.8% to 87.9% in the 2009–2013 birth cohorts, while the coverage of VarV_2_ increased from 31.8% for the 2009 birth cohort to 48.7% for the 2011 birth cohort. Higher coverage rates for both VarV_1_ and VarV_2_ were observed among resident children in relevant birth cohorts. The coverage rates of VarV_1_ and VarV_2_ were lower than those for the 1st and 2nd dose of MCV, which were above 95%. The proportion of children who were vaccinated with VarV_1_ at the recommended age increased from 34.6% for the 2009 birth cohort to 75.2% for the 2013 birth cohort, while the proportion of children who were vaccinated with VarV_2_ at the recommended age increased from 19.7% for the 2009 birth cohort to 48.7% for the 2011 birth cohort. *Conclusions*: Our study showed a rapid increasing VarV_2_ coverage of children, indicating a growing acceptance of the 2-doses VarV schedule among children’s caregivers and physicians after the new recommendation released. We highlighted the necessity for a 2-doses VarV vaccination school-entry requirement to achieve the high coverage of >90% and to eliminate disparities in coverage among sub-populations. We also recommended continuous monitoring of the VarV coverage via ZJIIS over time.

## 1. Introduction

Since 1998, varicella vaccine (VarV) has been licensed in Zhejiang Province, which is a developed province with a large population of 80 million located in East China. The 1-dose of VarV schedule for routine use in children aged ≥12 months was recommended by Zhejiang Provincial Center for Disease Control and Prevention (ZJCDC) since the approval of VarV. VarV is a category II (parent-pay) vaccine in China and the vaccination is voluntary. Since the schedule of 1-dose of VarV was recommended, VarV coverage increased rapidly and a substantial decrease in the incidence of varicella disease, varicella-related morbidity and mortality, and health care costs had been observed. Outbreaks of varicella, however, continued to occur in kindergartens or primary schools among children with high coverage of 1-dose VarV in Zhejiang Province. Some studies reported that approximately 15% of VarV recipients did not achieve protective levels of antibody and VarV induced immunity waned with time [1]. Besides, a large randomized trial indicated that the 2-doses VarV schedule was significantly more effective than the 1-dose VarV schedule [2]. In 2014, ZJCDC updated the recommendation regarding the use of VarV for varicella prevention. The latest recommendation includes a 3-pronged approach: (1) a routine 2-doses VarV schedule, with the 1st dose of VarV (VarV_1_) administrated at 12–15 months of age and the 2nd dose of VarV (VarV_2_) administrated at 3–4 years of age; (2) a VarV_2_ catch-up vaccination for children and adolescents aged ≤12 years who received VarV_1_ previously and without a history of varicella disease; (3) all persons aged ≥13 years without any evidence of immunity should be given 2 doses of VarV. According to the new recommendation, a minimum interval of 28 days between two doses of VarV is required.

At present, it is important to understand and continuously monitor the coverage of VarV among eligible age groups to guide future policy and intervention design. However, VarV coverage data of eligible children is not available as until now all the category II vaccines have not been included in the provincial level vaccination surveys. Therefore, little is known about the coverage of VarV by demographic characteristics such as gender or migration status. In fact, understanding of the coverage of VarV by these characteristics can provide valuable information for developing interventions tailored to subgroups with lower coverage.

The main objectives of this study included: (1) evaluating the coverage of VarV and exploring its determinants for children aged 2–6 years, and assessing the proportion of children who were vaccinated at the recommended age; (2) comparing the coverage of VarV with the coverage of measles containing vaccine (MCV), which had been included in the national immunization program (NIP).

## 2. Methods

### 2.1. Data Source

Data on VarV coverage were obtained from Zhejiang Provincial Immunization Information System (ZJIIS). ZJIIS, also known as the immunization registries, is a computerized, population-based system containing demographic and vaccination data for children aged <7 years living in Zhejiang Province since 2004 [3]. All vaccination clinics in Zhejiang Province must participate in ZJIIS. ZJIIS includes a client software deployed at each vaccination clinic and a database deployed in ZJCDC. The ZJIIS database consolidates data from different vaccination clinics through the Internet in real time and provides a tool for supporting effective vaccination strategies at the provider and program management level. Once any immunization clinic in Zhejiang Province is visited for the first time, children will be registered in ZJIIS with a unique identifier. Historical immunization information for migrant children is also included. Demographic information (such as name, date of birth, gender, address, phone number, immigration status) and vaccination records are collected by ZJIIS and these information will be updated in a day if there is any change.

Children aged 2–6 years (born from 1 January 2009 to 31 December 2013) and registered in ZJIIS were enrolled in this study. Appropriately anonymized individual records of target children were extracted from the ZJIIS database on 1 January 2016, including the VarV and MCV vaccination information. Children who were designated in ZJIIS as permanently inactive (*i.e.*, deceased) or “moved or gone elsewhere” were excluded.

### 2.2. Definitions

The VarV_1_ coverage was defined as the proportion of children who received VarV_1_, while the VarV_2_ coverage was defined as the proportion of children with VarV_1_ who received VarV_2_. Valid doses of VarV were defined as VarV_1_ administered no earlier than 4 days prior to the first birthday, VarV_2_ administered at least 28 days after VarV_1_, and either dose administered on the same day as or ≥4 weeks after any other live attenuated vaccine. All the invalid doses were considered as “unvaccinated” and were excluded from analysis.

The data were analyzed by birth cohort to evaluate the coverage of VarV at population or geography (by city) level, and for comparing the coverage of VarV and MCV. 12-month wide cohort was used for analysis among different cities, gender or immigration status. These cohorts were children born between 1 January, and 31 December 2009 for the 2009 birth cohort; children born between 1 January and 31 December 2010 for the 2010 birth cohort; children born between 1 January and 31 December 2011 for the 2011 birth cohort; children born between 1 January and 31 December 2012 for the 2012 birth cohort; children born between 1 January and 31 December 2013 for the 2013 birth cohort. The coverage of VarV_1_ was calculated for all the five cohorts while the coverage of VarV_2_ was calculated only for the 2009, 2010 and 2011 cohorts. Immigration status of children was classified as resident and migrant in this study. Migrant children included children from other provinces or from other cities of Zhejiang Province or from abroad as they were categorized in ZJIIS.

### 2.3. Analysis Strategy

The coverage rates of VarV_1_ and VarV_2_ of the relevant birth cohorts were calculated separately, as well as the proportion of children who were vaccinated at the recommended age 12–15 months for the VarV_1_ and 3–4 years for the VarV_2_. The VarV coverage was estimated among strata defined by cities, gender and immigration status. We evaluated separately the coverage of VarV_1_ and VarV_2_ in parallel to the coverage of the 1st and the 2nd dose MCV, which were scheduled at 8 months of age and 18 months of age, respectively. A *χ*^2^ test for trends in different birth cohorts was performed to evaluate whether there was a significant increase in coverage of VarV over time. We adopted the *χ*^2^ test to examine whether the coverage of VarV was significantly different across subgroup strata defined by gender or immigration status. We compared the coverage of MCV and VarV and evaluated the difference with the *χ*^2^ test. We performed all analysis with SPSS version 13.0 (SPSS Inc., Chicago, IL, USA) and at a significance level of 0.05.

### 2.4. Ethical Considerations

This study was approved by the Ethical Review Board of ZJCDC (No. T-043-R). All the data were anonymous when we exported them from ZJIIS and kept confidential without individual identifiers.

## 3. Results

### 3.1. Overall Coverage of VarV

A total of 3,028,222 children aged 2–6 years (born from 1 January 2009 to 31 December 2013) were enrolled in this study, of which 50.5% (1,527,818) were male and 60.0% (1,817,679) were migrant children. The VarV_1_ coverage for the 2009 birth cohort to the 2013 cohort was 84.8%, 86.8%, 88.6%, 88.5%, 87.9%, respectively. Across different birth cohorts, a significant increase in the coverage of VarV_1_ over time was observed in both the provincial and city level (*p* < 0.01). There were significant disparities in the coverage of VarV_1_ across the 10 cities for all the five birth cohorts. The coverage rates of VarV_1_ of all five cohorts in Wenzhou (WZ), Jiaxing (JX), Shaoxing (SX), and Jinhua (JH) were lower than the average rate of Zhejiang Province. The coverage rates of VarV_1_ of the 2012 birth cohort in WZ, JX, SX, JH, Quzhou (QZ), and Zhoushan (ZS) were lower than the average rate of Zhejiang Province. The coverage rates of VarV_1_ of the 2013 birth cohort in WZ, JX, JH, QZ, ZS were lower than the average rate of Zhejiang Province (Table 1).

A rapid increase of the VarV_2_ coverage was observed after the new VarV schedule was recommended in 2014. The VarV_2_ coverage increased significantly from 31.8% for the 2009 birth cohort to 48.7% for the 2011 birth cohort. Across the three birth cohorts, a significant increase in the VarV_2_ coverage over time was observed at both the provincial and city level (*p* < 0.01). There were significant disparities in VarV_2_ coverage across the 10 cities for the three birth cohorts. The coverage rates of VarV_2_ of the 2009 birth cohort in WZ, SX, JH, ZS were lower than the average rate of Zhejiang Province. The VarV_2_ coverage rates of the 2010 birth cohort in WZ, SX, JH, QZ, ZS were lower than the average rate of Zhejiang Province. The VarV_1_ coverage rates of the 2011 birth cohort in WZ, JH, QZ, ZS, and Taizhou (TZ) were lower than the average rate of Zhejiang Province (Table 2).

### 3.2. VarV Coverage across Subgroups

The VarV_1_ coverage was 87.6% for males and 87.2% for females in the 2009–2013 birth cohorts, and the VarV_2_ coverage was 38.3% for males and 37.3% for females in in the 2009–2011 birth cohorts. There was no significant difference in the VarV coverage between strata defined by gender in the 2009–2013 birth cohorts for the 1st dose, as for the 2nd dose (Table 3).

The VarV_1_ coverage was 96.3% for resident children and 81.5% for migrant children in the 2009–2013 birth cohorts, and the VarV_2_ coverage was 52.7% for resident children and 28.2% for migrant children in the 2009–2011 birth cohorts. There was significant difference in VarV_1_ and VarV_2_ coverage between strata defined by the immigration status of children. Higher coverage was observed in resident children in the 2009–2013 birth cohorts for VarV_1_, and similar pattern was also observed for VarV_2_ in the 2009–2011 birth cohorts (Table 4).

### 3.3. Coverage Difference between VarV and MCV

The VarV_1_ coverage increased from 84.8% for the 2009 birth cohort to 87.9% for the 2013 birth cohort, and it approached the coverage of the 1st dose of MCV (96.3%–97.8%). The VarV_2_ coverage of in the 2009–2011 birth cohorts was relatively consistent and it was significant lower than the 2nd dose coverage of MCV (95.5%–96.2%) (Table 5).

### 3.4. Age of Vaccinated Children

**Figure 1:**
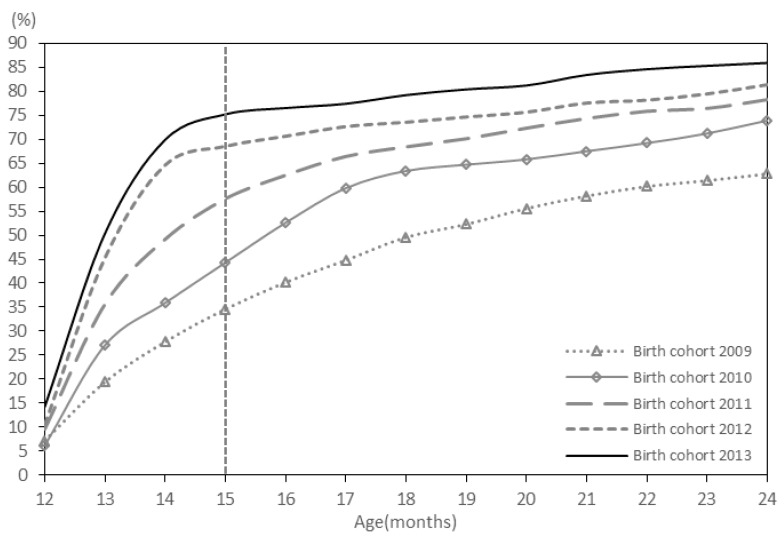
The coverage rate of VarV_1_ for children aged 2–6 years.

**Figure 2:**
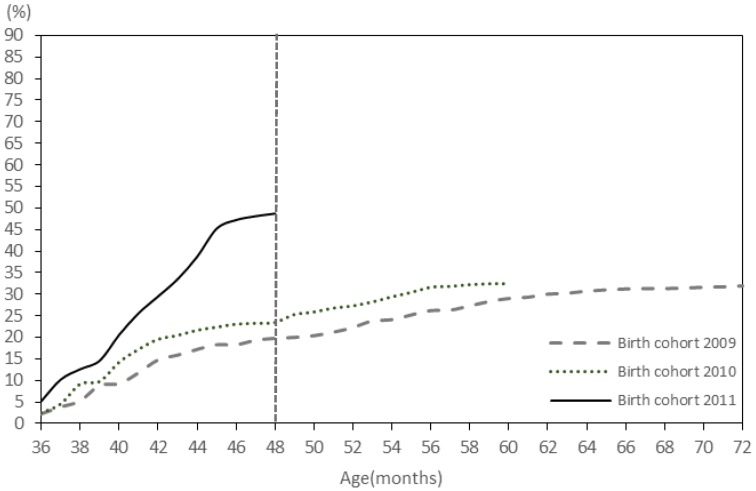
The coverage rate of VarV_2_ for children aged 4–6 years.

The proportions of children who received VarV_1_ at the recommended age at 12–15 months for the 2009 birth cohort to the 2013 cohort were 34.6%, 44.3%, 57.6%, 68.5%, 75.2%, respectively (Figure 1). The proportion of children who received VarV_2_ at the recommended age at 36–48 months (3–4 years) for the 2009 birth cohort to the 2011 cohort 2011 was 19.7%, 23.4%, 48.7%, respectively (Figure 2).

## 4. Discussion

Since the introduction of the 1-dose schedule of VarV, the VarV_1_ coverage increased significantly from the 2009 birth cohort to the 2013 birth cohort, approaching the goal of >90% for China’s NIP vaccines, but it was still lower than the VarV1 coverage in the USA [4], where the VarV_1_ coverage was 96.2% (95%CI: 96.2%–96.3%) for children aged ≥4 years in 2015. The high VarV_1_ coverage in the USA is mainly attributed to the 1-dose varicella vaccination school-entry requirement in place since 1999, which was changed to a 2-doses varicella vaccination requirement in 2006 [5].

Although the population-based incidence of varicella was not available due to the fact that until now varicella is not a notifiable disease in China, the national public health emergencies reporting system shows a steady decrease in the outbreaks of varicella in children aged ≤6 years in recent years.

Achieving a high 2-doses VarV coverage is critical for preventing varicella as the incremental vaccine effectiveness of 2-doses versus 1-dose of VarV was estimated as 63.6% [6]. Furthermore, even among the children who received VarV_1_ before, receipt the VarV_2_ also is important as the antibody level induced by VarV_1_ may wane with time since administration of VarV, which increases the risk of breakthrough varicella [7]. In this study, the VarV_2_ coverage was increasing rapidly. The results indicated the new recommendation of VarV vaccination was being quickly and widely accepted by physicians and caregivers and the VarV_2_ coverage for the 2011 birth cohort was much higher than that for the older children. These findings also demonstrated the relative success of the introduction of the 2-doses VarV schedule. However, the VarV_2_ coverage was still lower than that of VarV_1_, which potentially places unvaccinated children at risk for breakthrough varicella. Breakthrough varicella had been demonstrated as an impact factor in several prolonged outbreaks of varicella in high population intensity settings like schools or kindergartens, because breakthrough varicella is highly infectious and difficult to recognize [8].

Regional disparities in coverage were observed for both VarV_1_ and VarV_2_. Children from HZ, NB, TZ, LS had a significantly higher VarV_1_ coverage than that of children from other cities. Aside for the reason that VarV was a category II vaccine, this finding might be associated with the school-entry VarV_1_ vaccination requirement in these four cities, in addition to the school-entry requirement only for NIP vaccine in Zhejiang Province. We also found that HZ, NB, JX, LS had a higher VarV_2_ coverage than other cities. The VarV_2_ catch-up campaigns targeting the children aged 4–6 years with VarV_1_ which were conducted in 2015 in these four cities might explain the higher coverage of VarV_2_.

Children’s age remained a determinant of the VarV_2_ coverage, with a higher coverage seen for younger children in this study. The lower VarV_2_ coverage among older children might represent the failure to be vaccinated through a catch-up schedule. Another plausible explanation was that some of the older children with VarV_1_ might have had breakthrough varicella, but we could not verify this hypothesis from the available data. For children aged ≥3 years with only VarV_1_ and no history of varicella disease, the visits for the 4th oral polio vaccine at 4 years of age or the diphtheria and tetanus combined vaccine at 6 years of age may present an ideal time for VarV_2_ catch-up. Besides, a primary school-entry VarV_2_ requirement could be an efficient strategy to improve the coverage.

We did not find any difference in VarV_1_ and VarV_2_ coverage by children’s gender, which was consistent with our previous study [9]. However, we found meaningful disparities in VarV_1_ and VarV_2_ coverage by children’s immigration status. Lower coverage was observed in migrant children and it placed the migrant children at risk of varicella disease and of facilitating the transmission to other susceptible populations, as had been indicated for measles [10]. This result indicated that the utilization of vaccination services in migrant children was poorer than that in resident children, which could be associated with the lower demands for category II vaccines of the migrant caregivers and “service gap” that resulted from current allocation of public health resources. For migrant families, relative lower income and poor awareness of the importance of vaccination with category II vaccines are the main reasons for the lower demand for category II vaccines [9]. It means that a good income frees the household from the struggle of finding work to survive and would provide more spare time for caregivers to seek for public health service like immunization [11]. Besides, many of the migrant caregivers have a lower education level than residents, which results in a poorer understanding of the importance of vaccination of category II vaccines [11]. Furthermore, the public health resources, especially the vaccination nurses, are allocated according to the resident population with no consideration for the migrant population, which results in a “service gap”. That means the vaccination service capacity is sometimes inadequate in areas where the volume of migrant children is large or dense. As we know, people believe that medical workers are an authoritative source of information and vaccination nurses can also make more precise recommendations on a case-by-case basis to children’ caregivers when necessary. Unfortunately, most vaccination nurses working in vaccination clinics with a large scale migrant people do not have time to explain the importance of vaccinations to every caregivers. Therefore, the potential advantages of improving coverage posed through vaccination nurses’ recommendations are not efficiently utilized in these areas. School-entry vaccination requirement, including regulation of 2-doses VarV, had succeeded in improving the coverage, irrespective of children’s demographic characteristics such as the immigration status [12]. Therefore, the adoption and enforcement of a 2-doses VarV mandate in Zhejiang Province would not only be efficient in increasing the VarV_2_ coverage, but also address the coverage disparity between migrants and residents.

The coverage rates of VarV were significantly lower than those of MCV for both the 1st and the 2nd dose across all the birth cohorts. These findings were consistent with our previous survey conducted in Yiwu in 2014 [9] and demonstrates the importance of vaccines that are free of charge when setting for high coverage goals [13]. A higher VarV coverage could be expected if VarV were included in China’s NIP as a category I vaccine. The proportion rates of children who received vaccination at the recommended age of VarV_1_ and VarV_2_ were increased rapidly over time. The main reason for this was the wide application of ZJIIS in the routine vaccination service in Zhejiang Province. ZJIIS allows the generation of alerts that reminded providers that a vaccination is due or overdue. ZJIIS makes the remind/recall strategy more feasible and efficient, and ensures up-to-date vaccination. Additionally, the parents also had the access to their children’s vaccination records and information on the recommendation of vaccines via application programs derived from ZJIIS.

There are three limitations in our study. First, our analysis relied on the vaccination records of children registered in ZJIIS and the children who were not registered might have a lower coverage for all vaccines. As a result, we could have overestimated the coverage. Second, the ZJIIS data provides limited demographic information about the children and their families, so we could not explore other determinants of the coverage of VarV that were mentioned in the previous studies, such as caregivers’ education level, or socio-economic status. Third, we could not correlate the coverage of VarV with varicella incidence since varicella was not presently a notifiable disease in China.

## 5. Conclusions

Our study showed a rapid increase in the VarV_2_ coverage, indicating a growing acceptance of the 2-doses VarV schedule by children’s caregivers and physicians after the new recommendation was released. Further decline in the incidence of varicella disease can be expected as a higher 2-doses VarV vaccination coverage is achieved. Although our findings were encouraging, the coverage of VarV is still below the goal set for the NIP vaccine, placing school-age children at high risk of varicella or outbreaks. The findings of our study highlighted the necessity of school-entry VarV vaccination requirements to achieve high coverage (>90%) and to eliminate disparities in coverage among subgroups. We also recommended that the coverage of VarV be continuously monitored via ZJIIS to identify any challenges to the current VarV vaccination program.

## Figures and Tables

**Table 1:** Vaccination coverage of VarV_1_ of children aged 4–6 years in Zhejiang province, by city.

City ^#^	Coverage of VarV_1_										*p* ^§^
	Birth Cohort 2009 *		Birth Cohort 2010 *		Birth Cohort 2011 *		Birth Cohort 2012 *		Birth Cohort 2013 *		
	*n*/*N*	%	*n*/*N*	%	*n*/*N*	%	*n*/*N*	%	*n*/*N*	%	
HZ	78,821/87,784	89.8	85,349/94,079	90.7	97,873/106,238	92.1	111,272/121,288	91.7	104,740/115,606	90.6	<0.01
NB	80,385/87,082	92.3	85,937/91,708	93.7	95,951/100,381	95.6	103,641/108,063	95.9	98,724/102,965	95.9	<0.01
WZ	80,844/98,593	82.0	84,510/99,525	84.9	90,824/107,116	84.8	103,157/124,049	83.2	96,712/118,519	81.6	<0.01
JX	30,922/39,661	78.0	36,063/45,009	80.1	38,802/46,667	83.1	47,190/56,486	83.5	44,869/53,859	83.3	<0.01
SX	27,947/36,115	77.4	28,910/36,808	78.5	34,795/41,817	83.2	41,035/46,788	87.7	40,444/45,639	88.6	<0.01
JH	56,105/73,589	76.2	60,180/74,791	80.5	67,601/80,547	83.9	79,048/92,846	85.1	76,178/89,505	85.1	<0.01
QZ	15,509/18,220	85.1	13,909/16,055	86.6	16,661/19,090	87.3	19,462/22,368	87.0	18,398/21,217	86.7	<0.01
ZS	6094/7102	85.8	6257/7298	85.7	6572/7655	85.9	7591/9034	84.0	7062/8393	84.1	<0.01
TZ	60,269/67,403	89.4	61,264/67,565	90.7	65,859/72,124	91.3	73,101/81,595	89.6	68,500/77248	88.7	<0.01
LS	16,917/19,612	86.3	15,423/17,609	87.6	19,279/21,329	90.4	20,470/22,557	90.7	19,450/21,627	89.9	<0.01
Total	453,813/535,161	84.8	477,802/550,447	86.8	534,217/602,964	88.6	605,967/685,074	88.5	575,075/654,576	87.9	<0.01

**^#^** HZ: HangZhou; NB: NingBo; WZ: WenZhou; JX: JiaXing; SX: ShaoXing; JH: JinHua; QZ: QuZhou; ZS: ZhouShan; LS: LiShui; TZ: TaiZhou; * Difference in coverage among 10 cities was significant; **^§^** Two-tailed *p*-value of the *χ*^2^ test.

**Table 2:** Vaccination coverage of VarV2 of children aged 4–6 years in Zhejiang province, by city.

City ^#^	Coverage of VarV_2_						*p* ^§^
	Birth Cohort 2009 *		Birth Cohort 2010 *		Birth Cohort 2011 *		
	*n*/*N*	%	*n*/*N*	%	*n*/*N*	%	
HZ	32,701/87,784	37.3	39,378/94,079	41.9	58,038/106,238	54.6	<0.01
NB	52,631/87,082	60.4	42,102/91,708	45.9	75,853/100,381	75.6	<0.01
WZ	13,625/98,593	13.8	16,375/99,525	16.5	27,300/107,116	25.5	<0.01
JX	14,237/39,661	35.9	15,334/45,009	34.1	22,731/46,667	48.7	<0.01
SX	7492/36115	20.7	7643/36,808	20.8	21,674/41,817	51.8	<0.01
JH	12,808/73,589	17.4	21,307/74,791	28.5	33,361/80,547	41.4	<0.01
QZ	6142/18,220	33.7	4981/16,055	31.0	8575/19,090	44.9	<0.01
ZS	582/7102	8.2	1392/7298	19.1	3268/7655	42.7	<0.01
TZ	23,513/67,403	34.9	23,342/67,565	34.5	31,867/72,124	44.2	<0.01
LS	6348/19,612	32.4	6018/17,609	34.2	11,161/21,329	52.3	<0.01
Total	170,079/535,161	31.8	177,872/550,447	32.3	293,828/602,964	48.7	<0.01

**^#^** HZ: HangZhou; NB: NingBo; WZ: WenZhou; JX: JiaXing; SX: ShaoXing; JH: JinHua; QZ: QuZhou; ZS: ZhouShan; LS: LiShui; TZ: TaiZhou; * Difference in coverage among 10 cities was significant; **^§^** Two-tailed *p*-value of the *χ*^2^ test.

**Table 3:** Vaccination coverage of VarV for children born from 2009 to 2013 in Zhejiang province, by gender.

Birth Cohort	Coverage of VarV_1_					Coverage of VarV_2_				
	Male		Female		p *	Male		Female		p *
	*n/N*	%	*n/N*	%		*n/N*	%	*n/N*	%	
2009	228,808/269,186	85.0	225,008/265,975	84.6	>0.05	86,678/269,186	32.2	83,503/265,975	31.4	>0.05
2010	243,835/279,627	87.2	233,953/270,820	86.4	>0.05	91,158/279,627	32.6	86,636/270,820	32.0	>0.05
2011	271,539/305,100	89.0	262,687/297,864	88.2	>0.05	149,194/305,100	48.9	144,450/297,864	48.5	>0.05
2012	305,485/345,962	88.3	300,806/339,112	88.7	>0.05	-	-	-	-	-
2013	288,917/327,942	88.1	286,455/326,633	87.7	>0.05	-	-	-	-	-
Total	1,338,584/1,527,818	87.6	1,308,909/1,500,404	87.2	>0.05	327,030/853,913	38.3	314,589/834,659	37.7	>0.05

* Two-tailed *p*-value of the *χ*^2^ test.

**Table 4:** Vaccination coverage of VarV for children born from 2009 to 2013 in Zhejiang province, by immigration status.

Birth Cohort	Coverage of VarV_1_					Coverage of VarV_1_				
	Resident Children		Migrant Children		p *	Resident Children		Migrant Children		p *
	*n*/*N*	%	*n*/*N*	%		*n*/*N*	%	*n*/*N*	%	
2009	199,470/217,625	91.7	254,347/317,536	80.1	<0.01	98,418/217,625	45.2	71,763/317,536	22.6	<0.01
2010	208,419/219,120	95.1	269,369/331,327	81.3	<0.01	100,927/219,120	46.1	76,868/331,327	23.2	<0.01
2011	236,217/241,302	97.9	298,010/361,662	82.4	<0.01	157,659/241,302	65.3	135,985/361,662	37.6	<0.01
2012	267,962/271,974	98.5	338,329/413,100	81.9	<0.01	-	-	-	-	-
2013	253,823/260,521	97.4	321,548/394,054	81.6	<0.01	-	-	-	-	-
Total	1,165,891/1,210,542	96.3	1,481,602/1,817,679	81.5	<0.01	357,003/678,047	52.7	284,616/1,010,525	28.2	<0.01

* Two-tailed *p*-value of the *χ*^2^ test.

**Table 5:** Comparative assessment of vaccination coverage of VarV and MCV for children born from 2009 to 2013 in Zhejiang province.

Birth Cohort	Coverage of the 1st Dose (%)			Coverage of the 2nd Dose (%)		
	VarV	MCV	p *	VarV	MCV	p *
2009	84.8	96.3	<0.01	31.8	**95.5**	<0.01
2010	86.8	96.3	<0.01	32.3	96.2	<0.01
2011	88.6	97.8	<0.01	48.7	96.4	<0.01
2012	88.5	97.4	<0.01	-	-	-
2013	87.9	96.8	<0.01	-	-	-
Total	87.4	97.0	<0.01	38.0	96.0	<0.01

* Two-tailed *p*-value of the *χ*^2^ test.
